# Pterostilbene mitigates experimental pulmonary arterial hypertension by inhibiting endothelial-to-mesenchymal transition

**DOI:** 10.3389/fphar.2025.1621700

**Published:** 2025-06-25

**Authors:** Jie Wang, Yu Zhang, Junjun Liu, Fan Jiang, Xiaopei Cui, Weida Lu

**Affiliations:** ^1^ Department of Geriatric Medicine and Laboratory of Gerontology and Anti-Aging Research, Qilu Hospital of Shandong University, Jinan, Shandong, China; ^2^ Jinan Clinical Research Center for Geriatric Medicine, Qilu Hospital of Shandong University, Jinan, Shandong, China

**Keywords:** pterostilbene, pulmonary arterial hypertension, endothelial-to-mesenchymal transition, high mobility group AT-hook, SNAI, twist

## Abstract

**Background:**

The natural compound pterostilbene (PTE) has multiple cardiovascular protective effects. However, its effects on pulmonary arterial hypertension (PAH)-associated vascular remodeling remain to be elucidated. This study investigated the effects of PTE on monocrotaline (MCT)-induced PAH in rats *in vivo* and explored the underlying molecular mechanisms in human primary pulmonary arterial endothelial cells (hPAECs) *in vitro*.

**Methods:**

Experimental PAH was established by subcutaneous injection of MCT (50 mg/kg) in Sprague-Dawley rats, which were then randomly divided into vehicle or PTE (15 mg/kg via gavage) treatment groups. Endothelial-to-mesenchymal transition (EndMT) was modeled in hPAECs by treating with transforming growth factor-β, tumor necrosis factor-α, and interleukin-1β in combination.

**Results:**

In rats with MCT-induced PAH, administration of PTE resulted in a reduction in right ventricular systolic pressure, thereby alleviating right ventricular hypertrophy. This was accompanied by mitigation of the remodeling of pulmonary arteries. *In vitro*, genome-wide mRNA sequencing identified that PTE significantly downregulated the expression of high mobility group AT-hook 2 (HMGA2), a transcription factor involved in the pathogenesis of EndMT. Further, we demonstrated that PTE attenuated EndMT-related changes, including (1) reduced expression of the endothelial cell-specific markers platelet and endothelial cell adhesion molecule 1, and von Willebrand factor; (2) reduced nitric oxide production; and (3) increased expression of smooth muscle α-actin and other pro-fibrotic genes. Finally, we confirmed *in vivo* that PTE treatment reduced the expression of HMGA1/2 and Snai1/2 (markers of EndMT), and restored the expression of von Willebrand factor in the lungs of PAH rats.

**Conclusion:**

PTE mitigates MCT-induced PAH and vascular remodeling in rats, at least in part, by inhibiting HMGA-mediated EndMT, suggesting that PTE may be a useful complementary medicine in the treatment of PAH.

## 1 Introduction

Pterostilbene (PTE) is a stilbene compound originally isolated from the deciduous tree *Pterocarpus marsupium* native to India (Kim et al., 2020). The most common dietary sources of PTE include grapes and edible berries (Kim et al., 2020; Nagarajan et al., 2022). Like other plant-derived stilbenoids of the family (such as resveratrol and piceatannol), PTE has a broad range of biological activities, including antioxidant, anti-inflammatory, hypoglycemic, hypolipidemic, and anti-tumor effects (Akinwumi et al., 2018; Nagarajan et al., 2022). In the cardiovascular system, PTE has been shown to reduce ischemia-reperfusion-induced death of cardiomyocytes (Kosuru et al., 2018), and alleviate pathologic cardiac remodeling and contractile dysfunction induced by myocardial infarction or pulmonary arterial hypertension (PAH) (Dos Santos Lacerda et al., 2017; Lacerda et al., 2018). In apolipoprotein E-deficient mice, PTE treatment suppressed the development of atherosclerosis (Wang et al., 2019). Moreover, there was evidence suggesting that PTE had positive inotropic effects on pressure overload-challenged myocardium by regulating the expressions of antioxidant factors (e.g., glutathione-S-transferase and glutaredoxin) and Ca^2+^ handling proteins (e.g., phospholamban and sarcoplasmic reticulum Ca^2+^ ATPase) (Lacerda et al., 2020).

PAH is caused by the progressive remodeling of the pulmonary vasculature, characterized by thickening of both the tunica intima and tunica media of small arteries, leading to obstruction of the blood flow and increase in the vascular resistance (Humbert et al., 2008; Tuder, 2017). Endothelial cell dysfunction, smooth muscle hypertrophy, and aberrant proliferation of both endothelial and smooth muscle cells were all involved in the pathogenesis of PAH (Humbert et al., 2008; Tuder, 2017). In addition to these mechanisms, accumulating evidence has shown that endothelial-to-mesenchymal transition (EndMT), a pathological process analogous to epithelial-to-mesenchymal transition (EMT), may also have a critical role in the development of vascular remodeling in PAH (Ranchoux et al., 2015; Hopper et al., 2016; Tang et al., 2018). EndMT is a process in which endothelial cells undergo a shift of their phenotype from endothelium to smooth muscle- or myofibroblast-like cells. During EndMT, endothelial cells lose specific endothelial markers such as platelet and endothelial cell adhesion molecule 1 (PECAM-1) and vascular endothelial (VE)-cadherin, and progressively express smooth muscle markers like smooth muscle α-actin (α-SMA) and vimentin (Arciniegas et al., 2007; Gorelova et al., 2021). Simultaneously, endothelial cells acquire a strong migratory activity and become invasive (Arciniegas et al., 2007; Gorelova et al., 2021). It is clear that activation of the transforming growth factor (TGF)-β-Smad2/3 signaling pathway has a central role in promoting EndMT (Alvandi and Bischoff, 2021).

Previous studies demonstrated that PTE could inhibit migration and proliferation of vascular smooth muscle cells (Park et al., 2010; Lin et al., 2015), and improve the endothelial function by stimulating endothelial nitric oxide (NO) synthase (Park et al., 2015). Moreover, in alveolar epithelial cells, it was shown that PTE inhibited TGF-β-induced EMT (Peng et al., 2021). Currently, however, there is no direct information about the effects of PTE on PAH and associated vascular remodeling. In the present study, we provided evidence suggesting that PTE might mitigate experimental PAH, at least partly, by suppressing EndMT.

## 2 Materials and methods

### 2.1 Reagents

Monocrotaline (MCT) (#HY-N0750) was purchased from MedChemExpress (Shanghai, China). PTE (#ZT210301, purity >98%) was purchased from WUXI CENTURY BIO-ENGINEER Co. (Jiangsu, China). Recombinant proteins of human TGF-β (#CA59), tumor necrosis factor (TNF)-α (#C008) and interleukin (IL)-1β (#CG93) were from Novoprotein (Jiangsu, China). The following primary antibodies were used in Western blotting and/or immunofluorescence experiments: anti-PECAM-1 (#ab182981), anti-α-SMA (#ab32575), and anti-Snai1/2 (#ab180714) were from Abcam (Cambridge, United Kingdom); anti-von Willebrand factor(vWF) (#66682-1-Ig) was from Proteintech (Hubei, China); anti-Twist1 (#bs-2441R) was from Bioss (Beijing, China).

### 2.2 Animal studies

The use of experimental animals was approved by the Institutional Animal Ethics Committee of Shandong University Cheeloo College of Medicine (document #KYLL-2018ZM-636). *In vivo* studies were conducted in accordance with the National Institutes of Health Guide for the Care and Use of Laboratory Animals (NIH Publications No. 8023), and the Animals in Research: Reporting *In Vivo* Experiments (ARRIVE) guidelines. For *in vivo* therapeutic studies, the sample size was pre-determined for each group (n = 8). In accordance with our previous studies (Xu et al., 2021; Dai et al., 2024), the required minimum sample size in each group was predetermined to be n = 8, based on a presumed mean difference of >30% and a variance of 20%. Meanwhile, efforts were taken to reduce the number of animals used according to the 3Rs (Replacement, Reduction, Refinement) principle. Male Sprague-Dawley rats of Specific Pathogen-Free grade (8–10 weeks of age, weighing 200–250 g) were purchased from Charles River Laboratories (Beijing, China), and housed in an air-conditioned environment (non-Specific Pathogen-Free grade) with 12-h light/dark cycles. Animals were maintained on a standard chow diet and autoclaved tap water *ad libitum*. MCT-induced PAH was established as described (Xu et al., 2021; Dai et al., 2024). MCT was initially dissolved in 1 N HCl, followed by neutralization with 0.5 N NaOH. The stock solution was further diluted in normal saline. MCT was administered as a single bolus injection (50 mg/kg subcutaneously). PTE was mixed in 0.5% carboxymethylcellulose to a final concentration of 3 mg/mL and was administered at a dose of 15 mg/kg via gavage (performed at 9:00 AM every day). Three days after MCT injection, animals were randomly allocated to either vehicle or PTE treatment groups; the treatments were continued to day 28.

### 2.3 Hemodynamic measurements

Rats were anesthetized with 3% isoflurane and maintained with 1.5% isoflurane, using an anesthesia machine (model R500, RWD Life Science, Shenzhen, China) connected to a mechanical ventilator (model DW-3000C, Zhenghua Bio-Instrument, Huaibei, Anhui Province, China). Body temperature was monitored and maintained using a thermostat operating table (model EZ VET, Friends Honesty Life Sciences Company Limited, Beijing, China). Mechanical ventilation was applied by tracheotomy and endotracheal intubation, with a tidal volume of 2 mL per 100 g body weight, 60 times per min. An open-chest right ventricle catheterization procedure was performed as described previously (Xu et al., 2021). Right ventricular systolic pressure (RVSP) was recorded using a BL-420E biological data processing system (Techman Co. Ltd., Chengdu, Sichuan Province, China) (Supplementary Figure S1). To minimize the technical bias introduced during hemodynamic measurements, all of the procedures were standardized before experimentation, and were performed by a single researcher. Successful induction of PAH was verified by an elevated RVSP of >40 mmHg.

### 2.4 Tissue sample collection and processing

Rats were sacrificed by left atrial bleeding and perfused with cold saline at 100 mmHg via the left ventricle until tissues were pale. The heart was excised, and the right ventricle (RV) was carefully separated from the left ventricle (LV) and the interventricular septum (S) under a dissecting microscope. Each component was individually weighed using an electronic balance (FA1204B, Yueping, Shanghai, China). RV hypertrophy was assessed using the RV/(LV + S) ratio, a well-established index of RV hypertrophy (Stenmark et al., 2009). Part of the lung tissues were fixed in 4% paraformaldehyde overnight at 4°C; the remaining lung tissues were snap frozen in liquid nitrogen and stored at −80°C.

### 2.5 Cell culture and co-treatment with PTE

Human primary pulmonary arterial endothelial cells (hPAECs) (#HUM-iCell-a008) and human primary pulmonary arterial smooth muscle cells (hPASMCs) (#HUM-iCell-a009) were purchased from Cellverse (Shanghai, China), and cultured in complete ICell Primary Endothelial Cell Culture Medium (#PriMed-iCell-002 from Cellverse) and DMEM/F-12 (#11320033) medium supplemented with 10% FBS (Thermo Fisher Scientific, Waltham, MA, United States). Primary cells between passages 2 to 5 were used for experimentation. The mouse aortic vascular smooth muscle cell (MOVAS) was originally obtained from ATCC (#CRL-2797, Manassas, VA, United States), and cultured in high-glucose DMEM (#11965092 from Thermo Fisher) supplemented with 10% FBS. Cell cultures were maintained in a humidified atmosphere with 5% CO_2_ at 37°C. For PTE co-treatment experiments, the compound was dissolved in pure DMSO to various concentrations, and further diluted 1:200 in the culture medium. The final concentration of PTE used in hPAECs in culture was 20 μM. The same amount of DMSO was used as the vehicle control.

### 2.6 Cell viability and proliferation

Cell viability and proliferation were assessed using a colorimetric Enhanced Cell Counting Kit-8 (Beyotime, Beijing, China). Cells (3.5 × 10^3^ cells per well) were seeded in 96-well plates and cultured for up to 48 h. The optical density (OD) was obtained using an EMax Plus microplate reader (Molecular Devices, Sunnyvale, CA, United States) at 450 nm.

### 2.7 Measurement of NO release

The level of NO in hPAEC culture supernatants was determined using a colorimetric Nitric Oxide Assay Kit (#A012, Wuhan, Hubei Province, China), according to the manufacturer’s instructions. Absorbance at 550 nm was measured with a Sunrise plate reader (Tecan Group, Männedorf, Switzerland).

### 2.8 Real-time PCR

Total RNA was extracted using TRIzol Reagent (Thermo Fisher) and quantified with a NanoDrop 2000 spectrophotometer (Thermo Fisher). cDNA was synthesized using PrimeScript RT-PCR Kit (#RR037A from TaKaRa, Otsu, Shiga, Japan). The PCR reaction was performed using UltraSYBR Mixture kit (#CW0957M, from CoWin Biosciences, Taizhou, Jiangsu Province, China) with LightCycler 96 Instrument (from Roche, Basel, Switzerland). The following settings were applied: (1) 95°C for 10 min; (2) 40 cycles of 95°C for 16 s then 60°C for 1 min. Fold changes were determined using the 2^−ΔΔCt^ method, where ΔCt = Ct target gene–Ct housekeeping gene; ΔΔCt = ΔCt test sample–ΔCt calibrator (control) sample. The primer sequences used in real-time PCR were listed in Supplementary Table S1.

### 2.9 Western blot

Total proteins were extracted in a lysis buffer (#R0010) containing Protein Phosphatase Inhibitors cocktail (#P1260) and Protease Inhibitor Mixture (#P6730) (all purchased from Solarbio, Beijing, China). Proteins were separated by sodium dodecyl-sulfate-polyacrylamide gel electrophoresis and transferred to polyvinylidene fluoride membranes (from Merck KGaA, Darmstadt, Germany). The membranes were blocked with 5% nonfat milk and incubated overnight with primary antibodies at 4°C on an orbital shaker. Then the membranes were washed, incubated with horseradish peroxidase-conjugated secondary antibodies for 1–2 h at room temperature, and developed with SuperKine West Femto Maximum Sensitivity Substrate (#BMU102-CN, from Abbkine, Wuhan, Hubei Province, China). Images were captured using a gel imaging apparatus (Tanon 3500 Gel Imaging System, from Tanon Life Science, Shanghai, China). Band densitometry analysis was performed using ImageJ software.

### 2.10 Induction of EndMT *in vitro*

To induce EndMT in hPAECs, the cells were incubated with a combination of TGF-β (5 ng/mL), TNF-α (5 ng/mL), and IL-1β (0.1 ng/mL) as described previously (Good et al., 2015). In our preliminary experiments, we found that some early transcriptional events (such as the mRNA expression of EndMT-related transcriptional factors) were present after 3 days of stimulation, whereas the changes in phenotype-specific markers were not observed until 5 days of incubation. Therefore, different incubation durations (3 or 5 days) were applied to different experiments according to the specific endpoint measurements, which were indicated in the text. (Good et al., 2015; Gorelova et al., 2021).

### 2.11 Genome-wide mRNA sequencing

Cells were divided into 3 groups: Vehicle control, EndMT induced by TGF-β/TNF-α/IL-1β, and EndMT plus PTE co-treatment (20 µM). Three independent replicates were included for each group. Total RNA was extracted using TRIZOL Reagent (Thermo Fisher, Waltham, MA, United States) following the manufacturer’s instructions. RNA integrity was checked with an Agilent Bioanalyzer 2100 (Agilent Technologies, Santa Clara, CA, United States). Qualified total RNA was further purified by RNAClean XP Kit (Beckman Coulter, Brea, CA, United States) and RNase-Free DNase Set (QIAGEN, Germany). RNA concentration was determined using a NanoDrop ND-2000 spectrophotometer (Thermo Fisher). Library construction was performed using VAHTS Stranded mRNA-seq Library Prep Kit for Illumina (from Vazyme, Nanjing, Jiangsu Province, China). RNA sequencing was performed using a NextSeq Illumina550 platform (Illumina, San Diego, CA, United States) in a paired-end manner. RNA processing, library construction and sequencing services were provided by LC-BIO Co., Ltd. (Hangzhou, Zhejiang Province, China). The complete dataset of raw FPKM (fragments per kilo base per million mapped reads) values is publicly available at Mendeley Data (https://data.mendeley.com/datasets/4r2m5pyn6s/1; doi: 10.17632/4r2m5pyn6s.1).

### 2.12 Bioinformatics analysis

High-quality clean reads were obtained by filtering with Cutadapt (version 1.9) (https://cutadapt.readthedocs.io/en/stable/) and verified with FastQC (http://www.bioinformatics.babraham.ac.uk/projects/fastqc). Read alignment to GRCh38 human reference genome was performed using HISAT2 package (version 2.0.4) (https://daehwankimlab.github.io/hisat2). StringTie (version 1.3.4d) (http://ccb.jhu.edu/software/stringtie) was used for reading assembly and estimation of the relative expression levels (FPKM values). Differentially expressed genes (DEGs) were defined by the false discovery rate (*q* value) < 0.05 and fold change >2 or <0.5. Functional annotation and enrichment analysis for gene lists were carried out using DAVID platform (version 2021) (Sherman et al., 2022).

### 2.13 Histopathological examinations

Serial paraffin sections of 4-μm thickness were cut from the fixed lung tissue blocks. Sections were stained with routine hematoxylin and eosin staining (H&E). Digital images were captured using an Olympus BX43 microscope (Olympus Corporation, Tokyo, Japan). Morphometric measurements were performed using ImageJ software (NIH). The relative lumen area was defined as the percentage of the lumen area over the total vessel area. Histopathological analysis was performed in a blind manner.

### 2.14 Immunofluorescence

Cells were cultured on coverslips. After treatments, cells were fixed in 4% paraformaldehyde for 30 min, and permeabilized with 0.5% Triton X-100 for 15 min. The coverslips were blocked with 10% normal horse serum and incubated overnight with primary antibodies at 4°C. After washing, the coverslips were incubated with fluorophore-conjugated secondary antibodies and counterstained with DAPI. Images were taken using Olympus BX43 fluorescence microscope.

Tissue sections were deparaffinized and rehydrated. Antigen retrieval was performed by heating the slides in 10 mM sodium citrate buffer (pH 6.0) in a microwave oven for 3 min. Tissue sections were blocked with 1% bovine serum albumin at room temperature for 30 min, and incubated with primary antibodies at 4°C overnight. After washing, the sections were incubated with fluorophore-conjugated secondary antibodies at 37°C for 1 h. Digital images were taken using a Pannoramic Midi II slide scanner (from 3Dhistech, Budapest, Hungary). Semi-quantitative analysis of the fluorescent images was performed using ImageJ in a blind manner. Cellular expressions of PECAM-1 and α-SMA were classified as positive or negative by surveying all nuclei in a microscopic field. The expression level of vWF was assessed by measuring the mean fluorescence intensity (total fluorescence intensity/area) in cellularized areas, and expressed as continuous data. The expression of Snai1/2 in tissue sections was assessed by measuring the mean fluorescence intensity over the entire microscopic field. For each slide, 3 to 5 random fields were analyzed, and the data were averaged.

### 2.15 Cytoskeleton staining

Cells cultured on coverslips were fixed in 4% pre-cooled paraformaldehyde for 20 min on ice and permeabilized with 0.1% Triton X-100 for 10 min at room temperature. Cells were stained with 1× AbFluor™ 488-phalloidin working solution (#BMD00082 supplied from Abbkine Scientific, Wuhan, Hubei Province, China). Cells were counterstained with DAPI and photographed with Olympus BX43 fluorescence microscope.

### 2.16 Statistical analysis

Data were expressed as mean ± standard deviation (SD). Prism software (GraphPad, San Diego, CA, United States) was used for statistical analyses. For multi-group comparisons, one-way analysis of variance (ANOVA) followed by *post hoc* Tukey’s test was used. Two-tailed *P* values of <0.05 were considered to be statistically significant.

## 3 Results

### 3.1 PTE inhibited MCT-induced PAH and vascular remodeling

**FIGURE 1:**
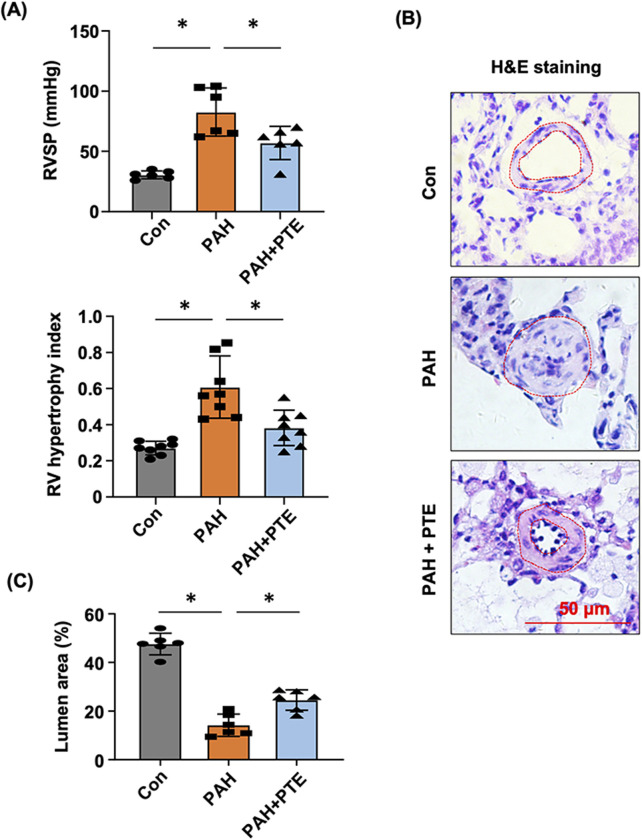
PTE inhibited vascular remodeling in MCT-induced PAH rats. **(A)** Effects of PTE on changes in RVSP (n = 6) and RV (n = 8) hypertrophy in PAH animals. **(B)** Representative histological images showed the effect of PTE on PAH-associated remodeling of small pulmonary arteries (outlined). **(C)** Quantitative data of the average percentage lumen area from different experimental groups. Data were expressed as mean ± standard deviation (SD). ^*^ *P* < 0.05, ANOVA. Each dot represented an independent sample.

In MCT-induced PAH models, RVSP was significantly elevated, which led to the development of RV hypertrophy (Figure 1A). Treatment with PTE ameliorated these changes. PAH was associated with pathologic remodeling of small pulmonary arteries (narrowing of the vessel lumen), while PTE treatment significantly inhibited the development of vascular remodeling (Figures 1B,C).

### 3.2 PTE had no effect on proliferation of pulmonary arterial smooth muscle cells

**FIGURE 2:**
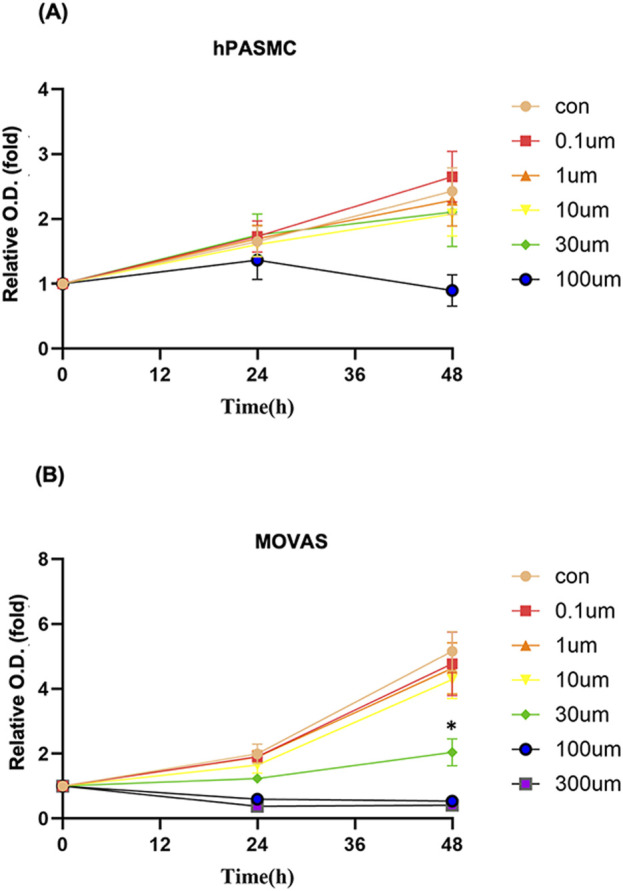
Effects of PTE at varying concentrations on the proliferation of hPASMC **(A)** and MOVAS **(B)**. Data were mean ± SD of five independent experiments respectively for hPASMC and three independent experiments respectively for MOVAS. ^*^ *P* < 0.05.

To clarify whether the beneficial effects of PTE on PAH were attributable to inhibition of smooth muscle cell proliferation, hPASMCs were treated with PTE of varying concentrations. The results showed that PTE from 0.1 μM to 30 μM had no effects on hPASMC proliferation (0–48 h), while PTE of 100 μM exhibited some cytotoxicity at 48 h (Figure 2A). The cytotoxicity of PTE over 100 μM was also confirmed in the MOVAS line (Figure 2B). PTE at 0.1–10 μM did not affect the proliferation of MOVAS, whereas PTE at 30 μM almost abolished the proliferation at 24 h compared to control (Con) cells (Figure 2B). Overall, the effects of PTE in smooth muscle cells lacked the typical sigmoidal concentration-response relationship. These data indicated that other mechanisms than the inhibition of smooth muscle cell proliferation were likely to be involved in mediating the beneficial effects of PTE on PAH.

### 3.3 PTE had no effect on cytokine-induced pro-inflammatory phenotype in hPAECs

**FIGURE 3:**
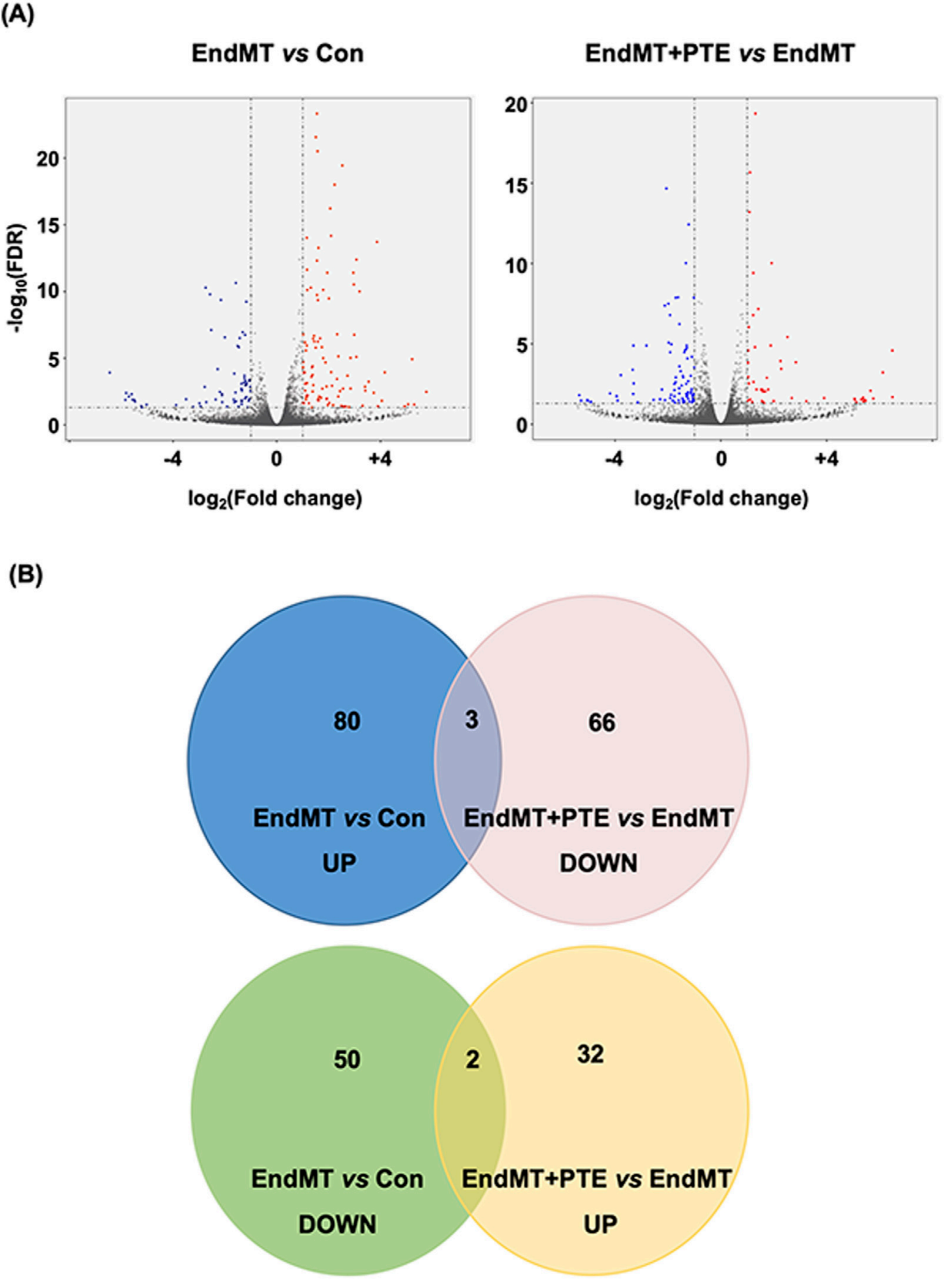
Profiles of differentially expressed genes identified by genome-wide mRNA sequencing in hPAECs. **(A)** Volcano plots showed the patterns of significantly upregulated (red) and downregulated (blue) genes between EndMT cells versus Con cells, and between EndMT cells with PTE (20 μM) treatment versus EndMT cells without PTE treatment. **(B)** Venn diagrams showed numbers of the differentially expressed genes between different experimental groups.

Attempting to delineate the mechanisms of the protective effects of PTE in the pulmonary vasculature, we induced inflammatory EndMT in hPAECs using a TGF-β/TNF-α/IL-1β cocktail as described (Good et al., 2015), and co-treated with PTE. Genome-wide mRNA sequencing was then performed to identify DEGs in response to the cytokine cocktail and PTE. Treatment with the cytokine cocktail for 3 days induced 135 DEGs, including 83 upregulations and 52 downregulations (Figures 3A,B; Supplementary Table S2). DAVID functional annotation analysis confirmed that the cytokine cocktail elicited a robust inflammatory response in hPAECs, for example, GO term “inflammatory response” (Benjamini *P* = 3.1 × 10^−11^); KEGG pathway “Cytokine-cytokine receptor interaction” (Benjamini *P* = 1.3 × 10^−9^); and UniProt Keywords “Inflammatory response” (Benjamini *P* = 3.9 × 10^−8^). Correspondingly, specific DEGs included upregulations of typical pro-inflammatory molecules involved in vascular inflammation, such as C-C motif chemokine ligand 2/monocyte chemoattractant protein 1 (*CCL2/MCP-1*), C-X3-C motif chemokine ligand 1 (*Fractalkine/CX3CL1*), intercellular adhesion molecule 1 (*ICAM1*), vascular cell adhesion molecule 1 (*VCAM1*), selectin E (*SELE*). In addition, the DEGs also included *IL1A* (IL-1α) gene and multiple *C-X-C* motif chemokine ligand genes (Supplementary Table S2). In addition, we found that the cytokine cocktail upregulated 1 marker of EMT/EndMT (snail family transcriptional repressor 1/SNAI1) and 3 pro-fibrotic genes (collagen type IV α1/COL4A1, collagen type IV α2/COL4A2, and TGF-β2/TGFB2). Of the 135 DEGs induced by the cytokine cocktail, only 15 (11%) were responsive to PTE co-treatment (Figure 3B). Moreover, functional annotation analysis revealed that the 103 PTE-induced DEGs (Figures 3A,B; Supplementary Table S3) were not significantly enriched in inflammation-related categories. Therefore, the protective effects of PTE against PAH were not directly attributable to inhibition of vascular inflammation. Interestingly, we observed that PTE significantly downregulated the expression of high mobility group AT-hook 2 (HMGA2) (Supplementary Table S3), a member of the HMGA family of architectural transcription factors with the ability to bind to AT-rich DNA sequences and to induce global changes in chromatin structure and transcription (Vignali and Marracci, 2020), which was implicated in the pathogenesis of EMT and EndMT (Belge et al., 2011; Hou et al., 2018; Li et al., 2023). Based on these results, we hypothesized that PTE might suppress EndMT in hPAECs by reducing HMGA2 expression.

### 3.4 PTE inhibited EndMT in hPAECs

**FIGURE 4:**
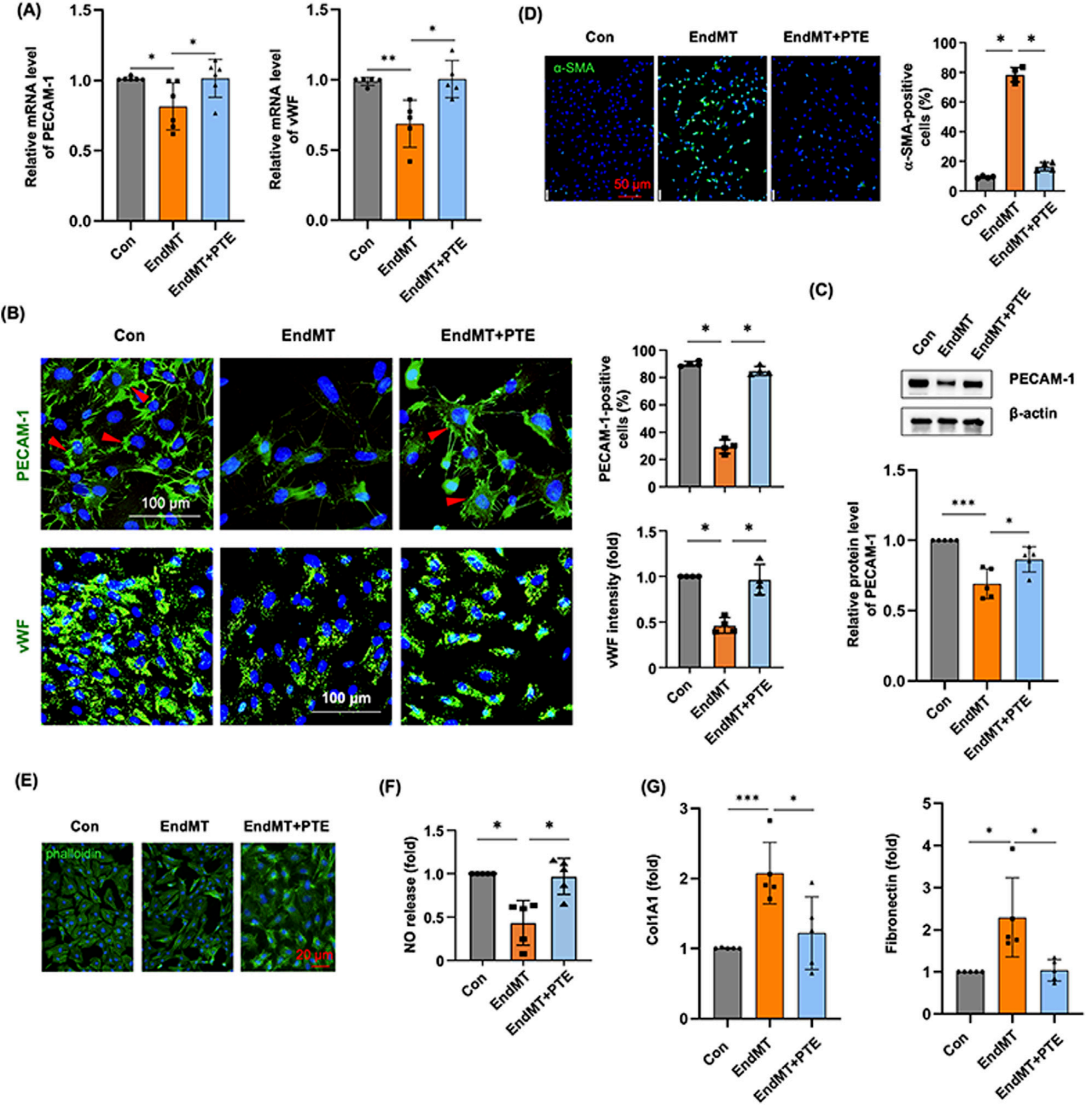
PTE inhibited the process of EndMT in hPAECs *in vitro*. **(A)** RT-qPCR results showed the effects of EndMT induction without and with PTE (20 μM) co-treatment on the expression of endothelial cell markers PECAM-1 and vWF. **(B)** Representative immunofluorescence images and quantitative data showed the effects of EndMT induction and PTE co-treatment on expressions of PECAM-1 and vWF (all in green color). Nuclei were counterstained with DAPI (blue). Only cells with typical surface expression of the PECAM-1 signal (red arrowheads) were counted as positive. **(C)** Western blot images from 3 independent experiments (Supplementary Figure S1) and the corresponding densitometry data showed the effects of EndMT induction and PTE co-treatment on the expression of PECAM-1. **(D)** Immunofluorescence images and quantitative data showed the effects of EndMT induction and PTE co-treatment on the expression of the smooth muscle cell marker α-SMA (green color). Nuclei were counterstained with DAPI. **(E)** Example fluorescence images of phalloidin staining of cytoskeleton showed the effects of EndMT induction and PTE co-treatment on the morphology of hPAECs in culture. Nuclei were counterstained with DAPI. **(F)** Effects of EndMT induction and PTE co-treatment on the release of NO in hPAECs. **(G)** PCR results showed the effects of EndMT induction and PTE co-treatment on the expression of Col1A1 and fibronectin in hPAECs. Data were mean ± SD. ^*^ *P* < 0.05, ^**^ *P* < 0.01, ^***^ *P* < 0.001, one-way ANOVA or unpaired *t*-test. Each dot represented an independent experiment.

Although we found that treating hPAECs with the cytokine cocktail for 3 days upregulated SNAI1 and several pro-fibrotic genes, the expression levels of specific endothelial markers such as PECAM-1 and vWF were not significantly changed. In order to induce a more robust EndMT response in hPAECs, we extended the incubation time to 5 days. Under this condition, we showed that EndMT induction reduced the mRNA levels of PECAM-1 and vWF, while PTE co-treatment blunted these changes (Figure 4A). PECAM-1 exhibited typical localization at the cell surface and cell-cell junctions in normal cells using immunofluorescence staining (Figure 4B). EndMT induction significantly decreased the number of PECAM-1-positive cells. In comparison, vWF exhibited clear cytosolic localization. EndMT also reduced the expression level of vWF. Semi-quantitative analyses showed that PTE co-treatment significantly inhibited the effects of EndMT (Figure 4B). To provide more evidence, we performed Western blot analysis on PECAM-1, and demonstrated similar results as immunofluorescence (Figure 4C; Supplementary Figure S2) (Note: the difference between Con and EndMT groups was only significant by unpaired *t*-test). The smooth muscle cell marker α-SMA was not expressed in normal hPAECs; EndMT induction significantly increased the number of α-SMA-positive cells, and this change was also ameliorated by PTE (Figure 4D). Normally cultured hPAECs exhibited the typical cobblestone-like endothelial cell morphology (Figure 4E). EndMT induction shifted the cell morphology to the spindle-like shape of smooth muscle cells, while PTE co-treatment partly restored the normal endothelial cell morphology (Figure 4E). To further confirm the de-differentiation status of EndMT hPAECs, we demonstrated that the NO production was reduced following EndMT induction and was restored by PTE treatment (Figure 4F). Finally, we demonstrated that EndMT induction was associated with a pro-fibrotic phenotype, as evidenced by the upregulated expressions of type I collagen (Col1A1) and fibronectin, while PTE treatment ameliorated the pro-fibrotic phenotype (Figure 4G).

### 3.5 PTE suppressed the HMGA-Snai/twist axis in hPAECs

**FIGURE 5:**
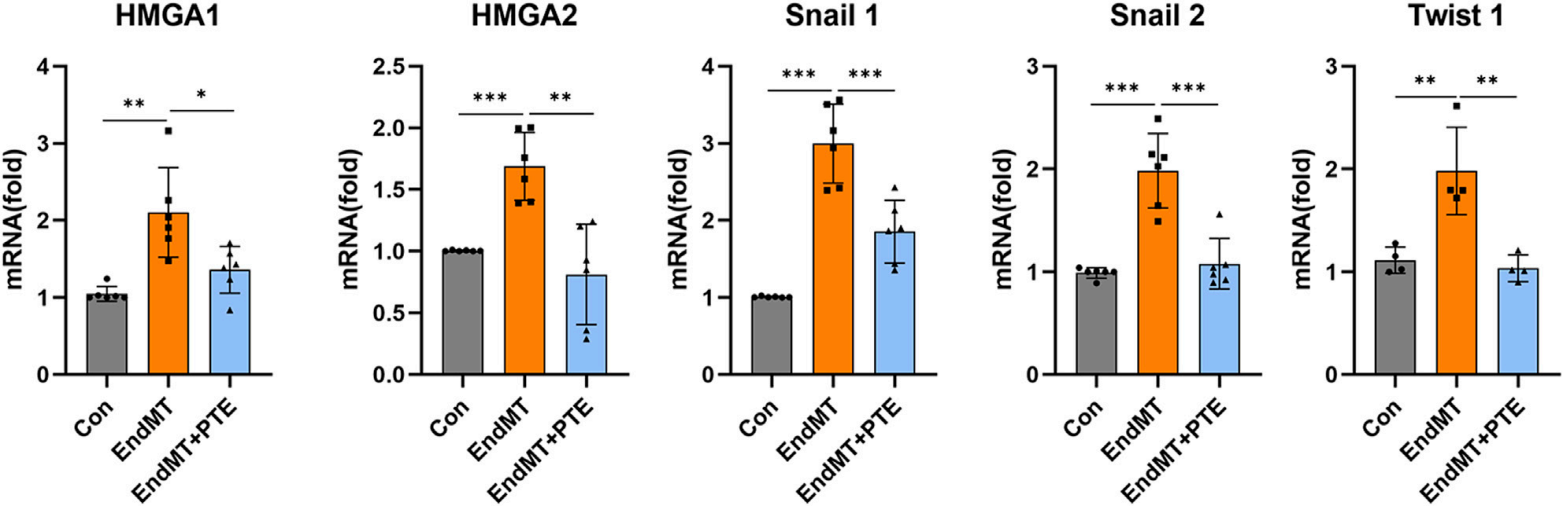
PTE suppressed the HMGA-Snai/Twist axis in hPAECs. The mRNA levels of various EndMT-related transcription-modulating factors in hPAECs were detected by qRT-PCR. The hPAECs were treated with TGF-β (10 ng/mL for 3 days) without and with PTE (20 μM) co-treatment. Data were mean ± SD. ^*^ *P* < 0.05, ^**^ *P* < 0.01, ^***^ *P* < 0.001, one-way ANOVA or unpaired *t*-test. Each dot represented an independent experiment.

The zinc finger transcription repressors Snai1 and Snai2 (snail family transcriptional repressor 1 and 2) (orthologues of the *SNAIL* and *SLUG* genes in *Drosophila*), and the basic helix-loop-helix domain-containing transcription factor Twist1 (twist family bHLH transcription factor 1) (orthologues of the *TWIST* gene in *Drosophila*) have been recognized to have pivotal roles in mediating EMT downstream of HMGA2-dependent transcriptional activation (Thuault et al., 2006; Thuault et al., 2008; Tan et al., 2012). In addition to HMGA2, there is evidence showing that HMGA1 is involved in promoting EndMT in pulmonary vascular endothelial cells by increasing Snai2 expression in PAH (Hopper et al., 2016). To further clarify whether the observed anti-EndMT effects of PTE were related to inhibition of the HMGA-Snail/Twist axis, we treated hPAECs with TGF-β (10 ng/mL for 3 days), and measured the expression levels of HMGA1, HMGA2, Snai1, Snai2 and Twist1 with PCR. As compared to control cells, HMGA1, HMGA2, Snai1, Snai2 and Twist1 were all significantly upregulated by TGF-β, while co-treatment with PTE reduced the expressions of these factors (Figure 5) (Note: the difference in HMGA1 expression between EndMT and EndMT + PTE groups was significant only by unpaired *t*-test).

### 3.6 *In vivo* evidence of reduced EndMT in the lungs with PTE treatment

**FIGURE 6:**
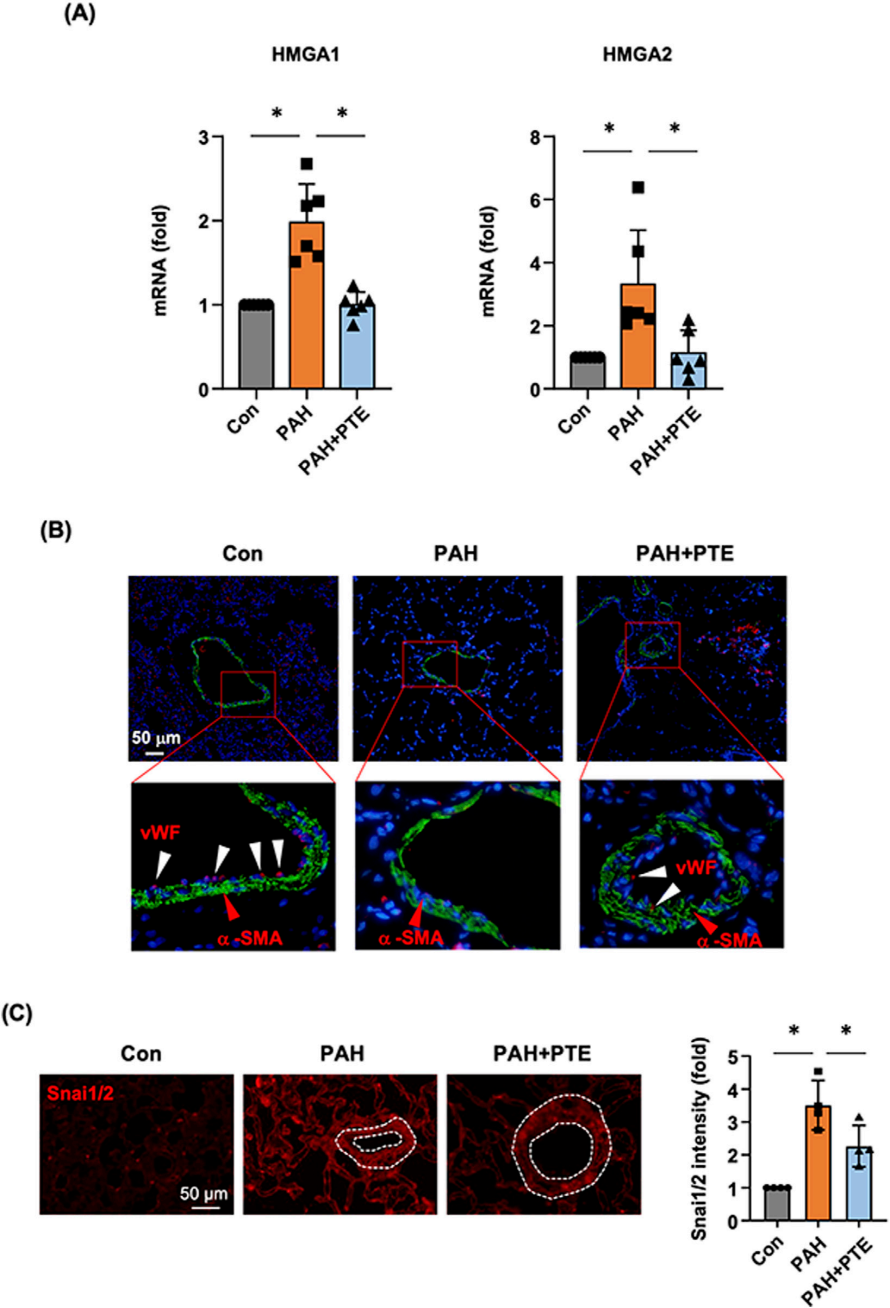
PTE treatment suppressed EndMT *in vivo* in the lungs from PAH models. **(A)** qRT-PCR results showed that PTE treatment reduced the pulmonary expression levels of HMGA1 and HMGA2 in PAH. **(B)** Representative images of immunofluorescence double labeling for α-SMA (green color) and vWF (red color), showed that in the normal lung, α-SMA exhibited typical localization in the medial layer of blood vessels (red arrowheads), while vWF was mainly located in the endothelial layer (white arrowheads). The expression of vWF was virtually absent in the blood vessels in PAH lungs, while PTE treatment partly restored vWF expression the endothelium. The data were an example from 4 independent tests showing similar results. **(C)** Representative immunofluorescence images and semi-quantitative data showed that Snai1/2 were widely expressed in the lung tissue (including blood vessels, outlined). Data were mean ± SD. *P < 0.05, one-way ANOVA. Each dot represented an independent sample.

To clarify whether PTE modulated the process of EndMT in the lungs during PAH, we measured the expression levels of HMGA1 and HMGA2 with real-time PCR. As shown in Figure 6A, the expressions of both HMGA1 and HMGA2 were significantly increased in PAH lungs, and these changes were blocked by the PTE treatment. Next, we performed immunofluorescence labeling for α-SMA and vWF. It was shown that in normal lung tissues, α-SMA exhibited typical expression in the medial layer of blood vessels, while vWF was mainly located in the endothelial layer (Figure 6B). vWF was also expressed in numerous scattered cells, which were presumably microvessel and alveolar endothelium. We observed that the expression of vWF was virtually absent in the blood vessels in PAH lungs, while PTE treatment partly restored vWF expression in the endothelium (Figure 6B). However, due to diminished vWF expression in the diseased vessels, it was technically difficult to accurately monitor the presence of α-SMA^+^/vWF^+^ double-positive (EndMT) endothelial cells. Nevertheless, we performed additional immunofluorescence labeling for Snai1/2. We showed that Snai1/2 were widely expressed in the lung tissue (including in blood vessels), which were upregulated in PAH. PTE treatment reduced the overall expression levels of Snai1/2 in PAH lungs (Figure 6C).

## 4 Discussion

Accumulating evidence suggests that EndMT fundamentally contributes to the pathogenesis of obstructive vascular remodeling associated with PAH (Gorelova et al., 2021). In the present study, we demonstrated that PTE treatment in rat models with MCT-induced PAH reduced the blood pressure in pulmonary circulation, and reduced the severity of lumen narrowing of small pulmonary arteries. However, our evidence did not indicate that modulation of the proliferation of smooth muscle cells had a major role in these beneficial effects; instead, we found evidence suggesting that PTE inhibited EndMT in pulmonary arterial endothelial cells, which could partly explain the inhibitory effects of PTE on PAH. Supporting our observations, a previous study showed that in MCT-treated rats without intervention, there was an increase of ∼40 mmHg in RVSP; treatment with PTE complexed with hydroxypropyl-β-cyclodextrin at varying doses reduced the RVSP increase by 15%–29% (Dos Santos Lacerda et al., 2017), although these authors did not examine the impact of PTE on pulmonary vascular remodeling. In addition, there was evidence suggesting that PTE may improve the contractility and enhance the antioxidative defense in stressed myocardium (Dos Santos Lacerda et al., 2017; Lacerda et al., 2020). Taken together, these pharmacological properties suggest that PTE may be a valuable complementary medicine in the treatment of PAH.

PTE has been shown to have anti-inflammatory activities. For example, in both murine and human macrophage cell lines, PTE repressed lipopolysaccharide-induced expressions of pro-inflammatory cytokines, including IL-6 and TNF-α (Yao et al., 2018; Patil et al., 2024). In vascular smooth muscle cells, PTE dampened activation of the pro-inflammatory transcription factor nuclear factor-κB (NF-κB) indirectly by stimulating the nuclear factor erythroid 2-related factor 2 (Nrf2) signaling pathway (Zou et al., 2024). In contrast to these results, the systemic biology analysis in our study failed to uncover a consistent anti-inflammatory activity of PTE in pulmonary arterial endothelial cells, probably because of differences in the pro-inflammatory stimulus used and the cell-specific nature of the PTE effects. However, our study cannot exclude possible contributions of the anti-inflammatory effects of PTE in other cell populations (especially immune cells) in the lungs, given the potential causal roles of local immuno-inflammatory reactions in the pathogenesis of PAH (Mercurio et al., 2021; Luo and Qiu, 2022; Wang et al., 2022).

**FIGURE 7:**
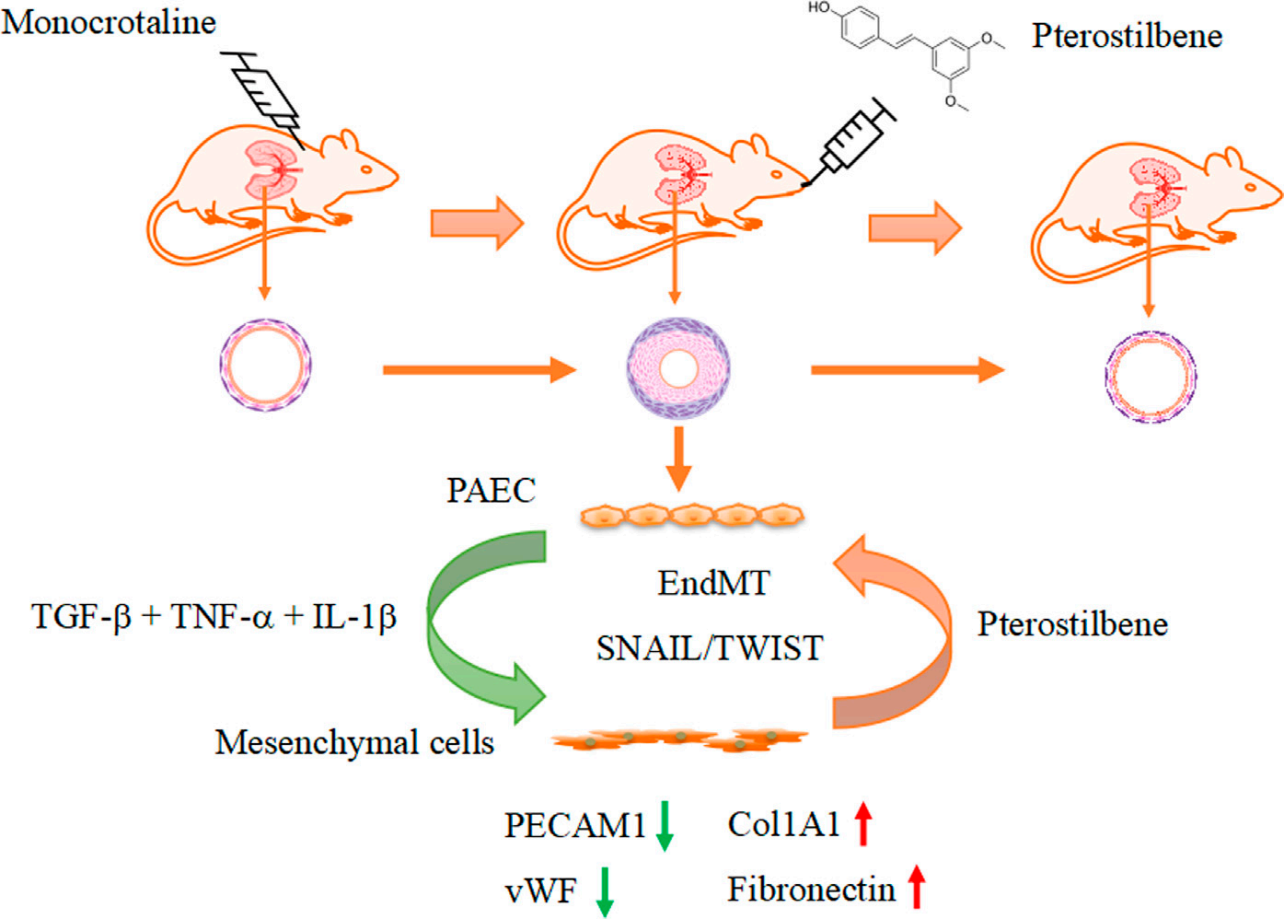
Summary of the effects of pterostilbene on PAH and associated vascular remodeling.

An interesting finding in the present study was that PTE inhibited the upregulation of Snai1/2 and Twist1 expressions and concomitantly blunted the induced EndMT process in pulmonary vascular endothelial cells. Both Snai1/2 (Hemavathy et al., 2000; Lin et al., 2014) and Twist1 (Ansieau et al., 2010) transcriptional modulators are key mediators of EMT during embryogenesis and tumorigenesis. The expression levels of Snai1/2 and Twist1 are controlled by HMGA2 (Thuault et al., 2006; Thuault et al., 2008; Tan et al., 2012). Consistently, we demonstrated that PTE similarly inhibited the expression of HMGA2. Likewise, all of HMGA2, Snai1 and Twist1 have been shown to be associated with the development of EndMT (Belge et al., 2011; Lee et al., 2018; Li et al., 2019; Li et al., 2023). Moreover, evidence indicates that HMGA1, another member of the high mobility group A proteins, is also implicated in mediating EndMT via Snai2 (Hopper et al., 2016). Hence, the inhibitory effect of PTE on HMGA1 expression may also underlie its anti-EndMT action. Supporting the *in vitro* data, we further confirmed that PTE reduced the expressions of HMGA1, HMGA2 and Snai1/2, and restored the expression of vWF in the lungs of PAH models *in vivo*. Therefore, based on these previous findings and our own results, we propose that PTE may inhibit EndMT in hPAECs by downregulating the HMGA1/2-Snai/Twist axis (Figure 7).

In human kidney proximal tubule epithelial cells (HK-2), Gu and coworkers provided evidence indicating that PTE inhibited the EMT response induced by high fructose exposure (Gu et al., 2019). Similarly, PTE appeared to inhibit TGF-β-induced EMT in rat kidney proximal tubular epithelial (NRK-52E) cells (Wang et al., 2020). Supporting these previous findings, here we first reported that PTE also exhibited inhibitory effects on EndMT in pulmonary vascular endothelial cells, likely by reducing HMGA1/2 expression. The mechanism(s) by which PTE regulates HMGA1/2 expression is not understood. It is known that HMGA2 transcription is stimulated by TGF-β (Thuault et al., 2006). Of note, emerging evidence indicates that PTE may suppress the development of tissue fibrosis partly by interfering with the TGF-β signaling pathway (Wang et al., 2024). Hence, it is reasonable to suggest that this action of PTE may also be involved in its anti-EndMT effects. Alternatively, HMGA1/2 expression is also governed by the Wnt/β-catenin pathway (Sgarra et al., 2018). This is not surprising since the Wnt/β-catenin pathway is indeed a downstream effector of TGF-β in mesenchymal cells (DiRenzo et al., 2016; Liu et al., 2019; Duan et al., 2020). Interestingly, in cancer epithelial cells, PTE appeared to reduce both basal and Wnt agonist-stimulated expression of β-catenin, leading to a decrease in cell proliferation (Paul et al., 2010). In addition to influencing β-catenin expression, PTE has also been shown to regulate the transcriptional activity of β-catenin (Zhang et al., 2011). Nevertheless, more experimental evidence is needed to verify the implications of these mechanisms in PTE-induced inhibition of EndMT in pulmonary vascular endothelial cells.

It is noted that there were several limitations in the present study. Firstly, the *in vivo* experiment only utilized a single PAH model, which may not adequately represent the heterogeneity observed in PAH patients. Future studies using different models (e.g., chronic hypoxia model and SU5416/hypoxia model) are needed to confirm the efficacy of PTE. In parallel, evaluations on the pharmacokinetics and long-term safety of PTE are also warranted. Secondly, only male animals were used for the PAH model. Adoption of this study design was because of the evidence suggesting that MCT-induced PAH was more stable in male animals as compared to females (Kasahara et al., 1997). Given that PAH is more common in female patients, it is important to further test the pre-clinical efficacy of PTE in female animal models. Thirdly our study cannot precisely confirm the endothelial origin of the mesenchymal-like cells *in vivo*. Hence, application of advanced techniques, such as cell lineage tracing and single-cell mRNA sequencing, may further specifically define the cellular target(s) of PTE in the lungs. Furthermore, it is interesting to test potential additive or synergistic effects between PTE and existing standard therapies.

## 5 Conclusion

Our results suggest that PTE may be a useful complementary medicine in the treatment of PAH, which suppresses the associated pathological vascular remodeling partly by inhibiting EndMT.

## Data Availability

The original mRNA sequencing data were deposited at Mendeley Data (https://data.mendeley.com/datasets/4r2m5pyn6s/1; doi: 10.17632/4r2m5pyn6s.1). Other data are available from the corresponding authors upon reasonable request.
