# Supplementary material for: Pterostilbene mitigates experimental pulmonary arterial hypertension by inhibiting endothelial-to-mesenchymal transition
Source: Front Pharmacol. 2025 Jun 25;16:1621700. doi: 10.3389/fphar.2025.1621700 (PMC12238021; doi:10.3389/fphar.2025.1621700)
Supplement: Supplementary file 3 [file Table1.docx]

**Supplementary Table S1.** **Primer sequences used in real-time PCR**

| **Gene** | **Forward** | **Reverse** |
| --- | --- | --- |
| ***human PECAM-1*** | TGACCCTTCTGCTCTGTTCAA | CTGAGGCTTGACGTGAGAGG |
| ***human vWF*** | CCTTGACCTCGGACCCTTATG | GATGCCCGTTCACACCACT |
| ***human Col1A1 (type I collagen)*** | TAAAGGGTCACCGTGGCTTC | GGGAGACCGTTGAGTCCATC |
| ***human fibronectin*** | AGCCGAGGTTTTAACTGCGA | CCCACTCGGTAAGTGTTCCC |
| ***human HMGA1*** | CAGCGAAGTGCCAACACCTAA | GTTGTGGTGGTTTTCCGGGT |
| ***human HMGA2*** | CAGCGCCTCAGAAGAGAGGA | TCTTCCCCTGGGTCTCTTAGG |
| ***human Snai1*** | GACCCCAATCGGAAGCCTAA | AGGGCTGCTGGAAGGTAAAC |
| ***human Snai2*** | GAGCATACAGCCCCATCACT | CTCACTCGCCCCAAAGATGA |
| ***human Twist1*** | GGACAGTGATTCCCAGACGG | CCTTTCAGTGGCTGATTGGC |
| ***rat HMGA1*** | CAGCCTTCGGTGAGTCCTG | TGGTAACTTTCCGCGTCTTGG |
| ***rat HMGA2*** | CACATCAGCCCAGGGACAA | TCTTGCTGCCTTTGGGTCTT |
