# Supplementary figures and images for: Pterostilbene mitigates experimental pulmonary arterial hypertension by inhibiting endothelial-to-mesenchymal transition
Source: Front Pharmacol. 2025 Jun 25;16:1621700. doi: 10.3389/fphar.2025.1621700 (PMC12238021; doi:10.3389/fphar.2025.1621700)

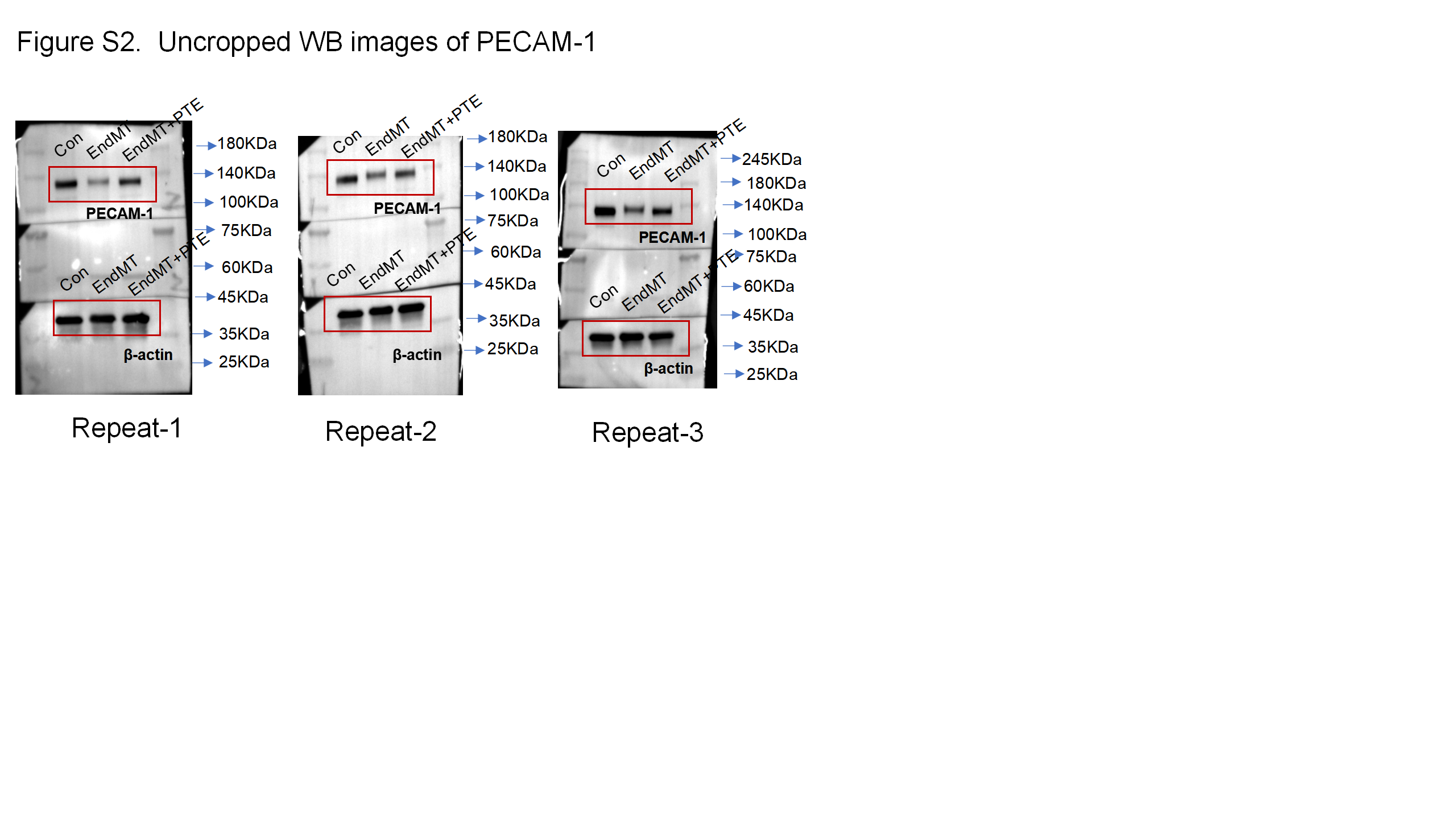

Supplement: Supplementary file 4 [file Image2.tif]

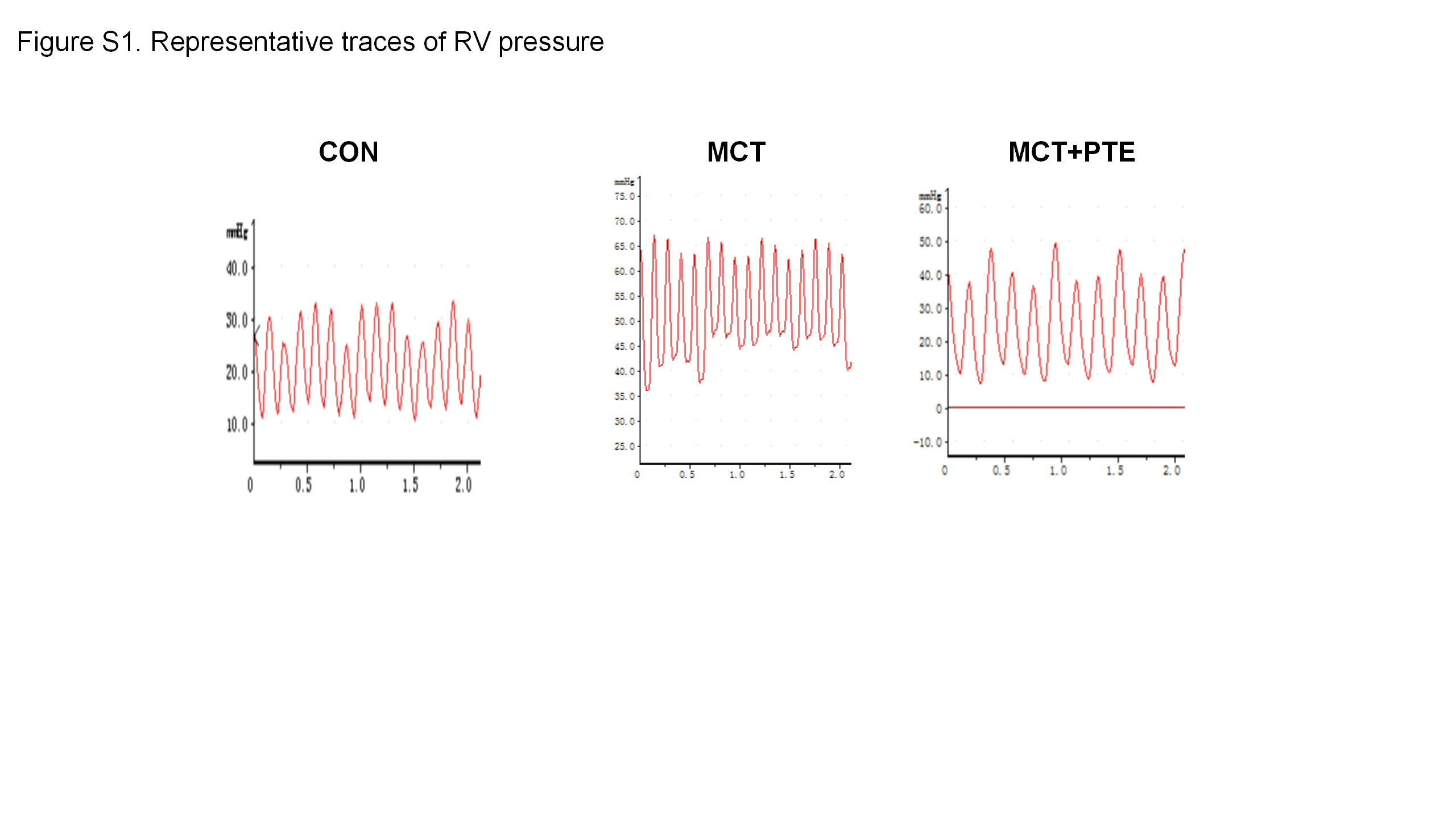

Supplement: Supplementary file 5 [file Image1.tif]
